# Supplementary material for: Interspecies radiative transition in warm and superdense plasma mixtures
Source: Nat Commun. 2020 Apr 24;11:1989. doi: 10.1038/s41467-020-15916-3 (PMC7181684; doi:10.1038/s41467-020-15916-3)
Supplement: Supplementary file 1 — Supplementary Information [file 41467_2020_15916_MOESM1_ESM.pdf]

Supplementary Information

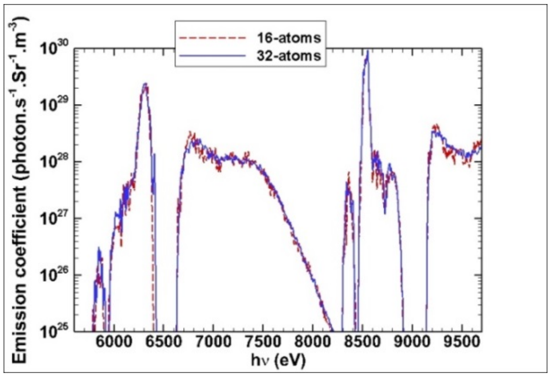

Supplementary Figure 1: Convergence testing results with respect to number of atoms in supercell. Comparison of emission spectra of Fe-Zn plasmas at ρ=1000 g cm^{-3} and kT=100 eV, using different number of atoms in super-cell for DFT calculations.

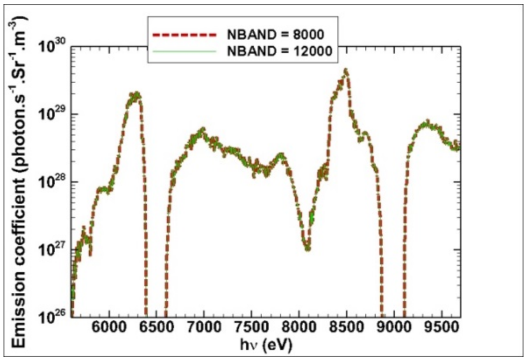

Supplementary Figure 2: Convergence testing results with respect to number of bands. Comparison of emission spectra of Fe-Zn plasmas at ρ=2000 g cm^{-3} and kT=50 eV (32-atom super-cell), using different number of bands in *ABINIT* calculations.

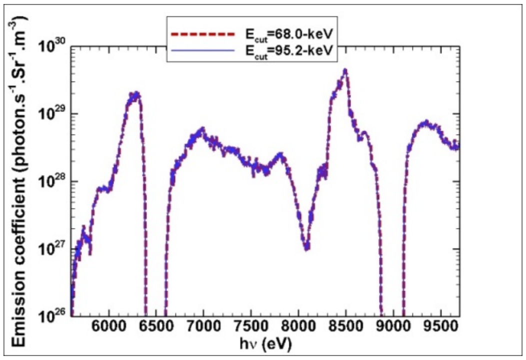

Supplementary Figure 3: Convergence testing results with respect to number of cut-off energies. Comparison of emission spectra of Fe-Zn plasmas at ρ=2000 g cm^{-3} and kT=50 eV (32-atom super-cell), using different plane-wave cut-off energies in *ABINIT* calculations.

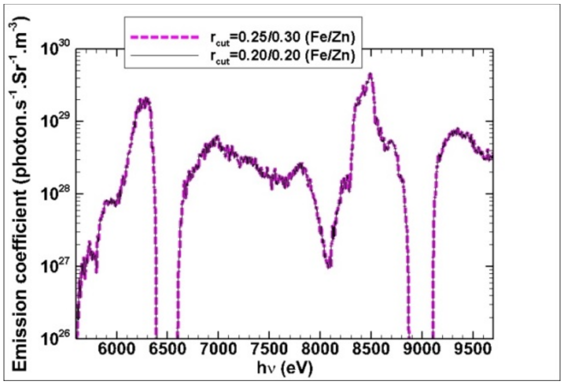

Supplementary Figure 4: Convergence testing results with respect to the cut-off radius of PAW pseudo-potentials. Comparison of emission spectra of Fe-Zn plasmas at ρ=2000 g cm^{-3} and kT=50 eV (32-atom super-cell), using different cut-off radius for all-electron PAW pseudo-potentials in *ABINIT* calculations.

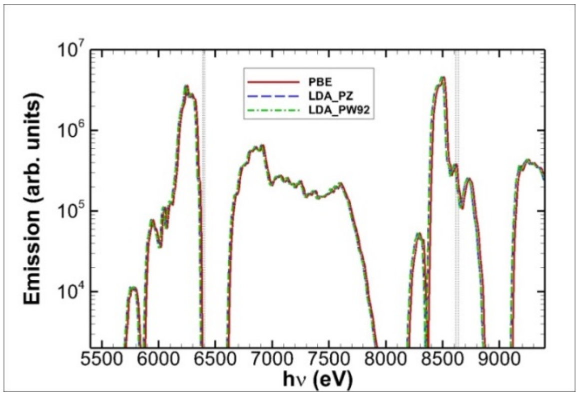

Supplementary Figure 5: Convergence testing results with respect to exchange-correlation functionals. Comparison of emission spectra of Fe-Zn plasmas at ρ=1500 g cm^{-3} and kT=50 eV (32-atom super-cell), using different exchange-correlation functionals in *ABINIT* calculations.

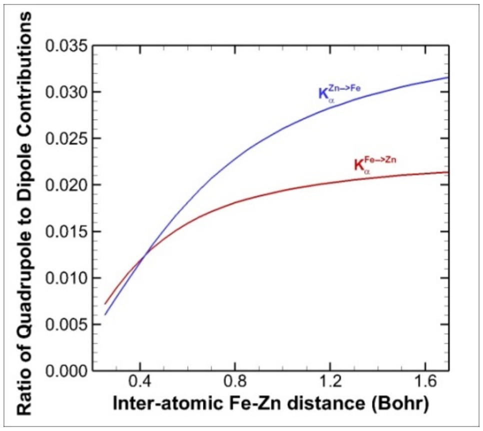

Supplementary Figure 6: Testing results of non-dipole contribution to inter-atomic K_α emission. The ratio of quadrupole to dipole contributions is plotted as a function of inter-atomic Fe-Zn distance.

Supplementary Note 1. CONVERGENCE TESTS

To obtain the converged emission spectra of super-dense plasmas, we have performed a variety of numerical tests on the number of atoms in super-cell, the number of bands, the plane-wave cut-off energy, and the cut-off radius of all-electron PAW pseudo-potentials. The convergence testing results are summarized in supplementary figures 1-4. In supplementary figure 1 we compare the results of emission spectra for Fe-Zn plasmas at ρ=1000 g cm^{-3} and kT=100 eV, using different number of atoms in super-cell for DFT calculations. It shows that the case of 32 atoms gives very similar result to the 16-atom case. Thus, the results presented in our paper had used 32-atom super-cell.

Supplementary figure 2 shows the convergence testing results on the number of bands used in our DFT calculations, for the emission spectra of dense Fe-Zn plasmas at ρ=2000 g cm^{-3} and kT=50 eV (32-atom super-cell). One can see that the calculation with 12000 bands give almost identical result as that of the 8000-band calculation. All of results presented in our paper were converged with a maximum of 18000 bands in *ABINIT* calculations (depending on the plasma density and temperature).

In supplementary figure 3 we plot the convergence testing results on the plane-wave cut-off energy used in our DFT calculations, for the emission spectra of dense Fe-Zn plasmas at ρ = 2000 g cm^{-3} and kT = 50 eV (32-atom super-cell). One can see that the calculation with $E_{cut} \approx$ 95.2-keV give almost identical result as that of $E_{cut} \approx$ 68-keV. All of results presented in our paper were converged with a maximum plane-wave cut-off energy varying from $E_{cut} \approx$ 40.8-keV to $E_{cut} \approx$ 95.2-keV in *ABINIT* calculations (depending on the plasma density and temperature).

Supplementary figure 4 shows the convergence testing results using different cut-off radius of all-electron PAW pseudo-potentials in our DFT calculations, for the emission spectra of dense Fe-Zn plasmas at ρ=2000 g cm^{-3} and kT=50 eV (32-atom super-cell). One can see that the calculation with $r_{cut}\approx$0.25/0.30 4 Bohr for Fe/Zn give almost identical result as that of $r_{cut}\approx$0.2 Bohr. All of results presented in our paper were converged with a minimum cut-off radius of $r_{cut}\approx$0.2 Bohr in *ABINIT* calculations.

Supplementary Note 2. EXCHANGE-CORRELATION FUNCTIONALS

To verify if our results are insensitive to the choice of exchange-correlation functionals, we have performed two additional DFT calculations with both LDA-PZ (Perdew-Zunger) and LDA-PW92 (Perdew-Wang-92). The results for Fe-Zn plasmas of ρ=1500 g cm^{-3} and kT=50-eV are shown by supplementary figure 5. One can see that these results are almost identical, except for a small (~10-15 eV) energy shift with each other. The inter-atomic K_α signals and their amplitude relative to the normal K_α emission are unchanged between LDA and PBE calculations.

Supplementary Note 3. NON-DIPOLE CONTRIBUTION

To examine the high-order electric quadrupole contribution to inter-atomic K_α emissions, we have computed its relative amplitude to that of dipole contribution with the independent-atom model. In supplementary figure 6, we plot the ratio of quadrupole to dipole contributions as a function of inter-atomic Fe-Zn distance. One clearly sees that as the inter-atomic distance increases the emitting entity gets bigger so that the quadrupole contribution rises. However, for the density range concerned, the maximum quadrupole term only gives less than ~3.2% of dipole contribution.
